# Alpha-Klotho is a novel predictor of treatment responsiveness in patients with heart failure

**DOI:** 10.1038/s41598-021-81517-9

**Published:** 2021-01-21

**Authors:** Manabu Taneike, Makoto Nishida, Kaori Nakanishi, Fusako Sera, Hidetaka Kioka, Ryohei Yamamoto, Tomohito Ohtani, Shungo Hikoso, Toshiki Moriyama, Yasushi Sakata, Keiko Yamauchi-Takihara

**Affiliations:** 1 Health and Counseling Center, Osaka University, 1-17 Machikaneyama, Toyonaka, Osaka 560-0043 Japan; 2 Department of Cardiovascular Medicine, Osaka University Graduate School of Medicine, 2-2 Yamadaoka, Suita, Osaka 565-0871 Japan

**Keywords:** Biomarkers, Cardiology

## Abstract

Heart failure is a major cause of death with an increasing population of elderly individuals. Several studies have demonstrated the involvement of soluble alpha-Klotho (sαKl) in various diseases. However, the correlation between sαKl and heart failure remains to be understood. The aim of this study is to investigate the levels and role of sαKl in patients with heart failure. Twenty-eight consecutive patients with acute heart failure (19 male, 9 female), admitted to the Osaka University Hospital from 2010 to 2018, were enrolled in this study. Mean NYHA score, left ventricular ejection fraction and BNP were 3.3, 17.0% and 588 pg/mL, respectively. SαKl significantly increased in heart failure patients. SαKl on admission were significantly higher in patients with heart failure who showed improvement after intensive treatment than that in patients who did not show improvement after the treatment. SαKl levels decreased significantly in patients who showed improvement. Interestingly, sαKl levels increased in male patients with heart failure, but not in female patients. Our data suggest that soluble αKl may be a novel biomarker for the responsiveness against treatment in patients with heart failure with reduced ejection fraction. Our findings may help developing a personalized therapy for different patients with heart failure.

## Introduction

Heart failure is the final stage in majority of the cardiac diseases that reduces the quality of life. Although currently used therapeutic strategies against heart failure have been carefully developed, the 5-year rate of mortality is still high and thus, heart failure is one of the leading causes of deaths globally^1^. Due to an aging society, there has been a rapid increase in the number of elderly patients with heart failure. Serum levels of proinflammatory cytokines, such as tumor necrosis factor (TNF)-α, are increased in patients with heart failure and show a correlation with the severity and prognosis of the disease^2^, suggesting that inflammation has an important role in the pathogenesis of heart failure. However, anti-TNF therapy could not improve primary trial end points^3^. Thus, there is an urgent need for the identification of a novel biomarker and therapeutic target for heart failure.

*Klotho* was discovered as a gene involved with anti-aging. The ablation of *Klotho* in mice manifests in a short life span and multiple aging phenotypes, such as skin atrophy, ectopic calcification, osteoporosis, atherosclerosis, and pulmonary emphysema^4^. *Klotho* encodes alpha-Klotho (αKl) that possesses a single-pass transmembrane domain and is expressed in the distal tubule of kidneys, parathyroid gland, and choroid plexus. Following studies revealed that αKl also regulates mineral metabolism and inflammation. Moreover, several studies have demonstrated the correlation between diseases and αKl^5–7^. A soluble form of αKl (sαKl) is detected in the serum and known to decrease with age^8,9^.

We have recently reported the protective effects of αKl by examining the serum levels of sαKl in healthy individuals. Smoking and psychological stress increases the levels of serum sαKl in men^10,11^. The increase in sαKl could preserve physical and mental conditions from the harmful effects of smoking and psychological stress.

Failing hearts show an increased expression level of Klotho mRNA; however, serum sαKl levels do not correlate with cardiac function or severity of heart failure^12,13^. The mechanism of the involvement of sαKl in heart failure remains to be fully understood. For example, patient data were mainly analyzed without considering age and sex, although serum levels of sαKl correlate with age and sex, as we previously reported^10^. This study aimed to understand the exact role of sαKl in patients with heart failure. Serum level of sαKl was significantly high, and decreased after treatment in the patients who showed improvement. Thus, sαKl may be a novel biomarker for the responsiveness to treatment in patients with heart failure.

## Results

### Biochemical characteristics of patients with heart failure

Table 1 shows the characteristics of the patients upon admission to the hospital. The mean NYHA score, EF and brain natriuretic peptide (BNP) concentration were 3.3, 17.0% and 588 pg/mL, respectively, indicating severe heart failure. Serum concentrations of BNP and C-reactive protein (CRP) markedly increased compared to their normal ranges applied at the Osaka University Hospital (BNP ≤ 40.0 pg/mL and CRP ≤ 0.20 mg/dL). Since patients with heart failure exhibit elevated serum levels of IL-6 and sαKl^12,14^, we determined IL-6 and sαKl levels in these patients. In accordance with previous reports, we found a marked increase in these levels. The mean serum concentrations of IL-6 and sαKl were 67.8 ± 40.0 pg/mL (normal range < 4.0 pg/mL) and 704.9 ± 42.6 pg/mL (average of age-matched control individuals: 531 ± 180 pg/mL^15^), respectively.

**Table 1:** Characteristics of patients with heart failure.

	On admission	After treatment
Age (years)	45.5 ± 2.6	45.6 ± 2.5
HR (/min)	83 ± 3^a^	75 ± 2^a^
sBP (mmHg)	93 ± 2	92 ± 3^c^
dBP (mmHg)	61 ± 2^b^	56 ± 2^d^
NYHA score	3.3 ± 0.1	2.7 ± 0.2
EF (%)	17.0 ± 1.3	23.2 ± 2.7^c^
BNP (pg/mL)	588 ± 67	299 ± 52
CRP (mg/dL)	1.26 ± 0.43	1.29 ± 0.47
IL-6 (pg/mL)	67.8 ± 40.0^a^	190.5 ± 170.8^c^
sαKl (pg/mL)	704.9 ± 42.6	608.9 ± 33.7
eGFR (mL/min/1.73 m^2^)	63.4 ± 3.4	74.2 ± 3.4
Data acquisition after admission (days)	6 ± 2	74 ± 11
Total hospital stay (days)	108 ± 15	

Patient characteristics were obtained on admission and after treatment (n = 28). Data are expressed as mean ± sem. Diagnosis: dilated cardiomyopathy n = 18, dilated hypertrophic cardiomyopathy n = 3, ischemic cardiomyopathy n = 3, fulminant myocarditis n = 3, hypertensive heart disease n = 1. Comorbidity: anemia n = 3, atrial fibrillation n = 4, dyslipidemia n = 7, diabetes n = 7, hyperuricemia n = 4, ventricular tachycardia n = 2.

BNP, brain natriuretic peptide; s/dBP, systolic/diastolic blood pressure; CRP, C-reactive protein; EF, left ventricular ejection fraction; eGFR, estimated glomerular filtration rate; HR, heart rate; IL-6, interleukin-6; NYHA, New York Heart Association functional classification; sαKl, soluble α-Klotho.

^a^n = 26, ^b^n = 25, ^c^n = 27, ^d^n = 20.

**Figure 1:**
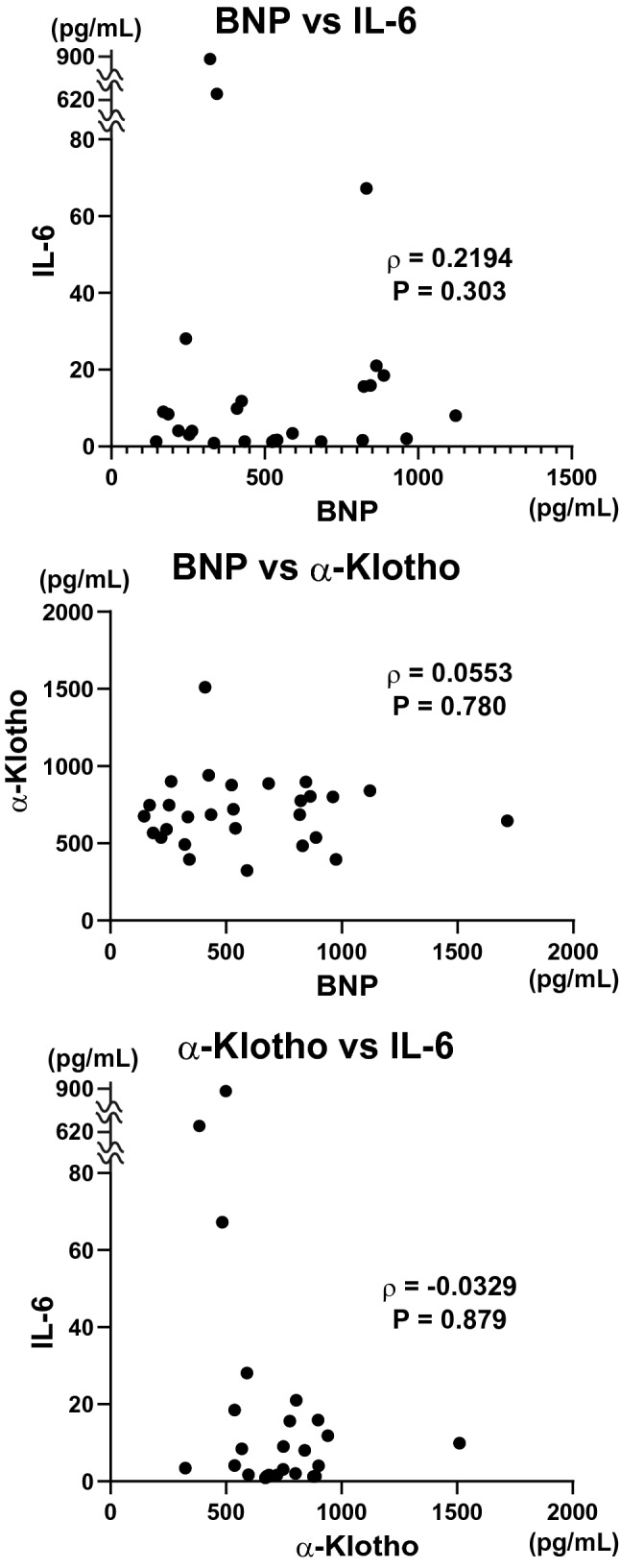
Correlation between serum BNP, IL-6, and α-Klotho levels. Serum BNP, IL-6, and α-Klotho levels were measured on admission. Spearman's correlation coefficient was used. BNP, brain natriuretic peptide; IL-6, interleukin-6.

To determine the correlation between these levels and severity of heart failure, we analyzed the correlation among serum levels of BNP, IL-6 and sαKl. There was no significant correlation between these biochemical parameters (Table 2 and Fig. 1). Although sαKl increased in heart failure patients, there was no correlation between sαKl and BNP.

**Table 2:** Correlation between serum BNP, IL-6, and α-Klotho levels.

	ρ	P value
BNP vs. IL-6	0.2194	0.303
BNP vs. α-Klotho	0.0553	0.780
IL-6 vs. α-Klotho	− 0.0329	0.879

Serum BNP, IL-6, and α-Klotho levels were measured on admission. Spearman's correlation coefficient was used.

BNP, brain natriuretic peptide; IL-6, interleukin-6.

### Responsiveness to treatment and sαKl levels

Although all patients received intensive care and medication for heart failure, some patients did not respond well. We classified the patients who showed improvement in their BNP level after the treatment as responders (n = 22) and who did not show improvement as non-responders (n = 6). As shown in Table 3, serum BNP level significantly improved and EF tended to improve in the responders. In addition, there was a tendency of a decrease in NYHA score in responders compared to non-responders after the treatment.

**Table 3:** NYHA score and BNP levels in patients on admission and after treatment.

	NYHA score		BNP (pg/mL)		EF (%)	
	On admission	After treatment	On admission	After treatment	On admission	After treatment
Responders (n = 22)	3.4 ± 0.2	3.0 ± 0.2	614.3 ± 82.3	184.2 ± 32.0*	17.1 ± 1.3	24.3 ± 3.4
Non-responders (n = 6)	3.2 ± 0.4	3.7 ± 0.3	491.4 ± 73.6	721.1 ± 79.9^†^	16.6 ± 3.9	19.3 ± 2.9

The New York Heart Association functional classification (NYHA) score, serum levels of brain natriuretic peptide (BNP) and left ventricular ejection fraction (EF) were determined in patients on admission and after treatment. The data after treatment were obtained 82.0 ± 13.7 and 43.2 ± 11.1 days after admission in responders and non-responders, respectively (P = 0.173). Data are expressed as mean ± sem. *P < 0.0001 compared to values on admission. ^†^P = 0.0003 compared to values of the responders. Repeated two-way ANOVA followed by Bonferroni’s post hoc test was used.

**Figure 2:**
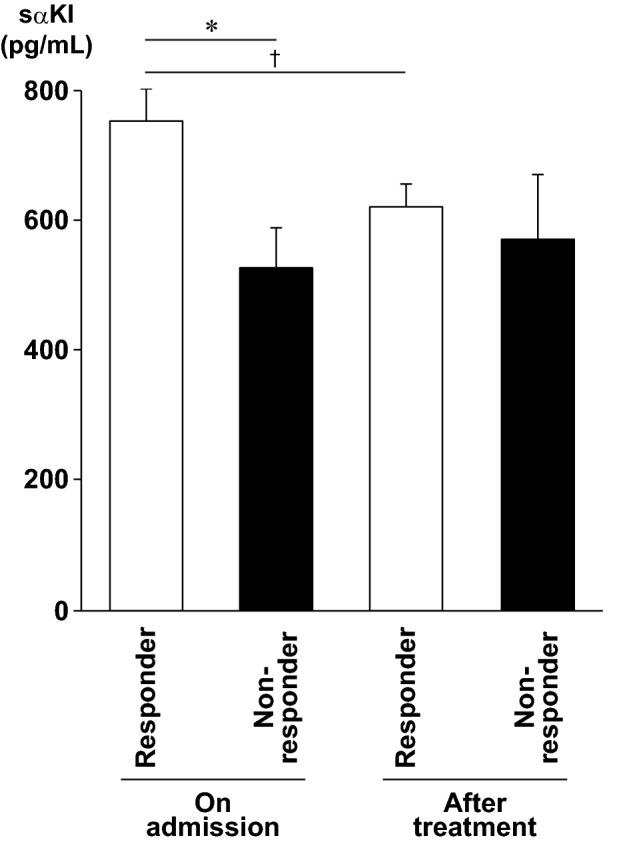
Serum α-Klotho levels in responders and non-responders. Serum α-Klotho levels were measured in patients with heart failure on admission and after treatment. Values are expressed as mean ± standard error of mean (sem). White and black bars indicate responders (n = 22) and non-responders (n = 6), respectively. *P = 0.0347. †P = 0.0027. Repeated two-way ANOVA followed by Bonferroni’s post hoc test was used.

We then compared serum levels of sαKl in both groups upon admission. As shown in Fig. 2, sαKl levels were significantly higher in responders than that in non-responders (753 ± 48 vs. 528 ± 61 pg/mL, P = 0.0347). Moreover, sαKl levels reduced in treated responders (619 ± 36 pg/mL, P = 0.0027), whereas there was no significant change in treated non-responders (572 ± 97 pg/mL).

### Gender difference in sαKl

To evaluate a gender difference in these parameters, we confirmed that there was no significant difference in NYHA (3.4 ± 0.2 vs. 3.3 ± 0.2, P = 0.958), age (44.6 ± 3.0 vs. 50.3 ± 5.1, P = 0.332) or any comorbidity between male and female patients on admission. Then, we analyzed IL-6 levels on admission in the patients, and observed substantial increased levels in both male and female patients (male controls: 1.9 ± 0.1 pg/mL; male patients: 87.6 ± 54.0 pg/mL; female controls: 1.1 ± 0.1 pg/mL; female patients: 14.0 ± 8.4 pg/mL).

**Figure 3:**
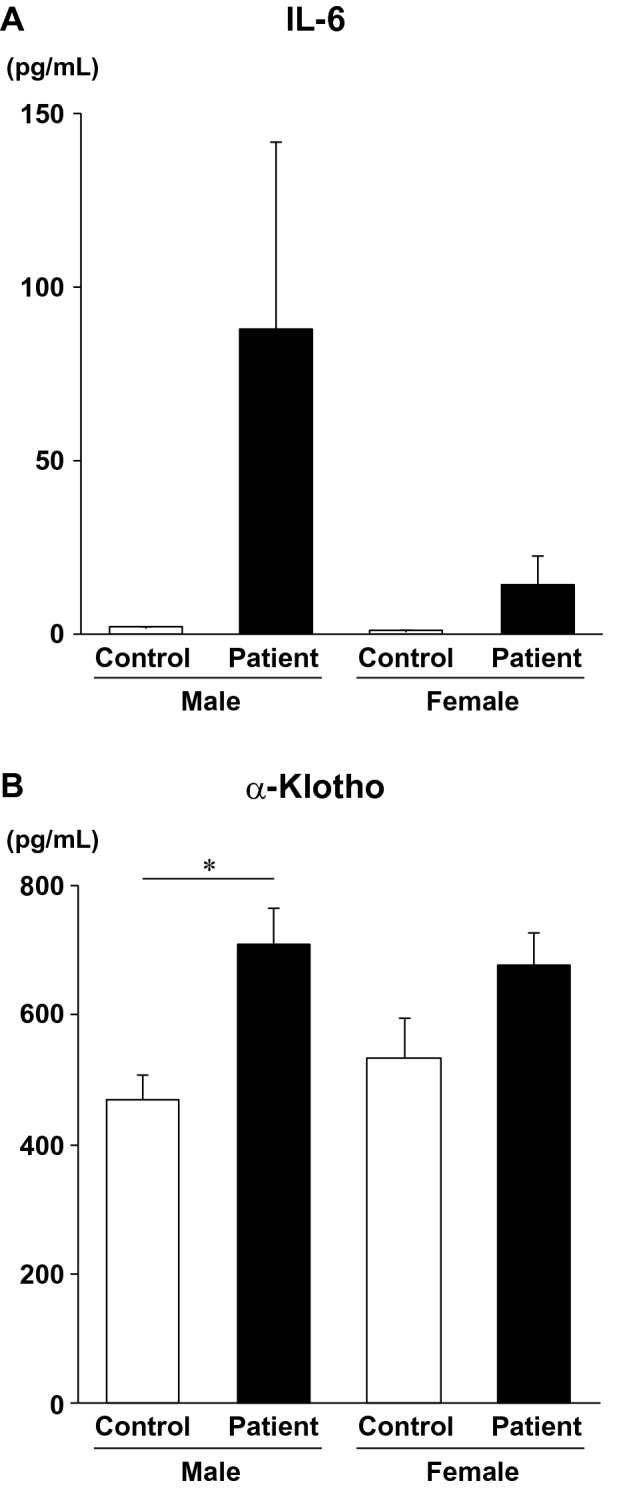
Serum IL-6 and α-Klotho levels in male and female patients. Serum IL-6 and α-Klotho levels were measured in healthy age-matched individuals (Control) and patients (Patient) with heart failure. Values are expressed as mean ± sem. White and black bars indicate controls and patients, respectively. *P = 0.0039. Non-repeated two-way analysis of variance (ANOVA) followed by Tukey’s post hoc test was used.

We reported that smoking affects serum levels of sαKl differently in males and females^15^. Thus, we next analyzed serum sαKl concentrations on admission of each gender (Fig. 3). There was a significant increase in serum sαKl level in male patients (473.6 ± 39.6 vs. 717.7 ± 58.3 pg/mL, P = 0.0039), however female patients did not show a significant increase (533.4 ± 60.7 vs. 677.8 ± 48.2 pg/mL).

## Discussion

Recent various studies show an important pathophysiological role of inflammation in the development and progression of heart failure. Serum levels of sαKl change in an age-dependent manner^8^ and are associated with anti-inflammatory effects^16^. In this study, we analyzed the correlation between sαKl levels and heart failure. Although serum concentration of sαKl markedly increased in patients with heart failure and decreased upon successful treatment, sαKl levels did not correlate with BNP as well as logBNP (data not shown). This is in accordance with a previous study that shows no correlation between sαKl and severity of heart failure^12^. On the other hands, a negative correlation between sαKl and BNP had been reported in the heart failure patients with preserved EF (15). Klotho is predominantly expressed in the kidney and diseased heart tissue^12^, but not in cardiac myocytes^4,17,18^. Although Klotho is a transmembrane protein, its extracellular domain is released from the cell surface and has multiple renal and extrarenal functions. The protective role of sαKl in the murine heart was reported and that Klotho deficiency induced cardiac hypertrophy^18^. Systemically circulating Klotho protects the heart from stress-induced pathological cardiac remodeling and fibrosis^19,20^. Thus, sαKl levels might indicate the status of the heart in patients with heart failure.

Serum levels of sαKl markedly increase by smoking and psychological stress in healthy men^10,11^. This increase in sαKl could be attributed to compensation against the negative effects of smoking, such as systemic inflammation. Similarly, there is a possibility that sαKl is produced as a compensatory response and protects the heart during heart failure by acting as a suppressor of inflammation. Thus, patients who respond to stimuli during heart failure and produce sαKl may have a better chance to ameliorate symptoms.

We evaluated the ratio of IL-6 to sαKl and found that the ratio was higher in non-responder group than in responder group after treatment (1.22 ± 1.08 vs. 0.00974 ± 0.00217, P = 0.0148 analysed by repeated two-way ANOVA followed by Bonferroni’s post hoc test), although there was no difference on admission, suggesting that sαKl was not sufficiently produced to regulate inflammation in non-responder group.

The relative risk of developing cardiovascular diseases after smoking is higher in women than that in men, suggesting women have higher sensitivity to the toxic effects of smoking^21^. Men and women have different sαKl responses to stress, such as smoking^15^; only men exhibit a positive correlation of sαKl levels with smoking status. In this study, similar to the smoking, males showed enhanced sαKl response to heart failure than females. This could be attributed to the poor prognosis of female patients with heart failure. Thus, as compared to male patients, female patients with heart failure require more strict intensive care. However, the non-responders in this study consisted of 5 males and 1 female, suggesting that the reduced responsiveness to treatment may not be only due to the sex of the individual. Furthermore, since female patients with heart failure did not comprise the majority of non-responders, that indicates the presence of other protective mechanisms apart from sαKl in females. On the other hand, it is difficult to be excluded that the smaller sample size (n = 9) could be one of possibilities for the non-significance in serum sα-Kl of female patients.

It is well known that serum level of BNP is a powerful tool for the evaluation of severity and prediction of prognosis for heart failure patients^22^. However, it is still not possible to apply a BNP-guided treatment to all patients with heart failure because of the heterogeneous etiology and patient background. Considering the significance of inflammation in the development of heart failure, sαKl could be an additional factor to determine a prognosis of the disease. sαKl might play a supplemental role to support treatment based on BNP for those patients.

This study is associated with several limitations. First, the study cohort was comprised of a small population; future studies will require an increased number of patients. Second, two out of six non-responders were patients with fulminant myocarditis. Thus, the pathogenesis of heart failure may affect the dynamics of sαKl levels in the non-responders. Third, circulating IL-6 increases during chronic heart failure (CHF) and are higher in patients with severe CHF than that in patients with mild CHF^23^. However, we did not observe a significant difference in the serum levels of IL-6 between heart failure patients and control individuals statistically, although they were markedly increased in male and female patients. This could be because of very low cardiac function and heterogenic etiology of the study subjects. Especially, patients who had fulminant myocarditis were all male. That might be the reason why male patients showed very high level of IL-6 with a large variation.

In conclusion, we have identified sαKl as a novel biomarker for the responsiveness of treatment in heart failure patients with reduced EF. Serum levels of sαKl were different in male and female patients with heart failure. These findings might help develop personalized therapeutic strategies for patients with heart failure.

## Methods

### Study subjects

The study subjects comprised 28 consecutive patients with acute heart failure who admitted to the Osaka University Hospital from 2010 to 2018 (19 males, 9 females; 24–80 years old). All patients suffered from heart failure with reduced ejection fraction (EF) owing to dilated cardiomyopathy, including idiopathic dilated cardiomyopathy, dilated hypertrophic cardiomyopathy, and ischemic cardiomyopathy (n = 25), and fulminant myocarditis (n = 3). Inclusion criteria were left ventricular EF ≤ 35% and estimated glomerular filtration rate (eGFR) ≥ 30 mL/min/1.73 m^2^ (0.739 times for female). It is known that the serum level of BNP is relatively higher and that of soluble alpha-Klotho is lower^24,25^ in patients with renal dysfunction compared to healthy controls. To minimize the effect of renal dysfunction on the analyses in this study, we excluded patients with severely reduced eGFR and kidney failure, CKD stage G4 and 5, respectively^26,27^. All patients were intensively treated using catecholamine, left ventricular assist devices, and etc., according to prevailing guidelines. Clinical data, including patient characteristics, laboratory data, and echocardiograms were obtained on admission.

The control subjects were chosen from individuals who underwent health examination at the Osaka University Health and Counseling Center (total 7332 persons). The criteria for the healthy control subjects were no declaration for past disease history, medication, smoking history or any symptom based on health questionnaire. The serum level of soluble alpha-Klotho was measured in randomly selected 115 subjects of the healthy controls. The age-matched controls in this study were randomly selected from the control subjects (19 males, 9 females).

This study was performed in accordance with the ethical guidelines for clinical research of the Ministry of Health, Labor and Welfare and the Ministry of Education, Culture, Sports, Science and Technology of Japan. All the protocols used in this study were approved by the ethics committees of the Osaka University Hospital and Health and Counseling Center, Osaka University. Written informed consent was obtained from all the individuals prior to participation.

### Biochemical characteristics

The New York Heart Association functional classification (NYHA) score was evaluated as follows: 1 = class I, 2 = class II, 3 = class III, and 4 = class IV. Serum was collected from individuals after overnight fasting on admission and after treatment and kept at ≤  − 80 °C until further use. Serum levels of interleukin (IL)-6 and sαKl were measured by using a chemiluminescent enzyme immunoassay (Fujirebio Inc., Tokyo, Japan) and a sandwich enzyme-linked immunoassay (Immuno-Biological Labs, Takasaki, Japan) according to the manufacturer’s instructions, respectively^10^.

### Statistical analysis

All statistical analyses including a test for normal distribution were performed using GraphPad Prism 8 (GraphPad Software, La Jolla, California). Data are expressed as mean ± standard error mean (sem) regardless of normality. Four-group comparisons were analyzed using non-repeated two-way analysis of variance (ANOVA) followed by Tukey’s post hoc test or repeated two-way ANOVA followed by Bonferroni’s post hoc test. Spearman's correlation coefficient was used to analyze non-parametric differences. P < 0.05 was considered statistically significant.
